# Neopeltolide and its synthetic derivatives: a promising new class of anticancer agents

**DOI:** 10.3389/fphar.2023.1206334

**Published:** 2023-06-06

**Authors:** Sheila I. Peña-Corona, Héctor Hernández-Parra, Sergio A. Bernal-Chávez, Néstor Mendoza-Muñoz, Alejandra Romero-Montero, María Luisa Del Prado-Audelo, Hernán Cortés, Dilek Arslan Ateşşahin, Solomon Habtemariam, Zainab M. Almarhoon, Ahmad Faizal Abdull Razis, Babagana Modu, Javad Sharifi-Rad, Gerardo Leyva-Gómez

**Affiliations:** ^1^ Departamento de Farmacia, Facultad de Química, Universidad Nacional Autónoma de México, Ciudad de México, Mexico; ^2^ Departamento de Farmacología, Centro de Investigación y de Estudios Avanzados del Instituto Politécnico Nacional, Ciudad de México, Mexico; ^3^ Facultad de Ciencias Químicas, Universidad de Colima, Coquimatlán, Mexico; ^4^ Escuela de Ingeniería y Ciencias, Tecnologico de Monterrey, Monterrey, Mexico; ^5^ Laboratorio de Medicina Genómica, Departamento de Genómica, Instituto Nacional de Rehabilitación Luis Guillermo Ibarra Ibarra, Ciudad de México, Mexico; ^6^ Department of Plant and Animal Production, Baskil Vocational School, Fırat University, Elazıg, Türkiye; ^7^ Pharmacognosy Research and Herbal Analysis Services UK, University of Greenwich, London, Kent, United Kingdom; ^8^ Department of Chemistry, College of Science, King Saud University, Riyadh, Saudi Arabia; ^9^ Department of Food Science, Faculty of Food Science and Technology, Universiti Putra Malaysia, Selangor, Malaysia; ^10^ Natural Medicines and Products Research Laboratory, Institute of Bioscience, Universiti Putra Malaysia, Selangor, Malaysia; ^11^ Department of Biochemistry, Faculty of Science, University of Maiduguri, Maiduguri, Nigeria; ^12^ Facultad de Medicina, Universidad del Azuay, Cuenca, Ecuador

**Keywords:** neopeltolide, marine-derived macrolide, cytotoxic, natural antiproliferative drugs, cancer

## Abstract

Being the first or second cause of death worldwide, cancer represents the most significant clinical, social, and financial burden of any human illness. Despite recent progresses in cancer diagnosis and management, traditional cancer chemotherapies have shown several adverse side effects and loss of potency due to increased resistance. As a result, one of the current approaches is on with the search of bioactive anticancer compounds from natural sources. Neopeltolide is a marine-derived macrolide isolated from deep-water sponges collected off Jamaica’s north coast. Its mechanism of action is still under research but represents a potentially promising novel drug for cancer therapy. In this review, we first illustrate the general structural characterization of neopeltolide, the semi-synthetic derivatives, and current medical applications. In addition, we reviewed its anticancer properties, primarily based on *in vitro* studies, and the possible clinical trials. Finally, we summarize the recent progress in the mechanism of antitumor action of neopeltolide. According to the information presented, we identified two principal challenges in the research, i) the effective dose which acts neopeltolide as an anticancer compound, and ii) to unequivocally establish the mechanism of action by which the compound exerts its antiproliferative effect.

## 1 Introduction

The decline in cancer mortality over the past three decades reflects progress in cancer-preventing measures, diagnosis, and disease management (Santucci et al., 2020). However, cancer remains to be the main cause of disease burden worldwide (Sung et al., 2021; Kocarnik et al., 2022). Current estimates show that cancer is the first or second most frequent cause of death before the age of 70 in 112 of 183 nations (WHO, 2020). It is also projected to become a primary cause of morbidity and mortality in all countries in the very near future (Bray et al., 2012; Kocarnik et al., 2022). Consequently, there is a continued call to double the global effort to research on developing novel chemicals with anticancer properties.

Cancer patients are treated through surgery, chemotherapy, radiation therapy, immunotherapy, and targeted therapy (among others), depending on the kind and step of the disease (Benjamin, 2014; Min et al., 2014; Baudino, 2015; Ghosh, 2019). The chemotherapeutic option includes various synthetic drugs which are endowed with numerous side effects and limited efficacies. For example, cisplatin is one of the most widely administered medications for treating various cancers, but its application is limited by drug resistance and organ toxicity (Ferrarelli, 2018; Ghosh, 2019). This again justifies the need to developing, studying, and characterizing new natural anticancer drugs with low toxicity and improved efficacy.

About a third of the most widely sold drugs trace their origin to natural products or have been formulated from structures obtained from natural sources (Qi and Ma, 2011). In addition, some drugs or drug leads of natural origin are more active than synthetic anticancer agents (Napolitano et al., 2009). One good example of natural anticancer drug lead is neopeltolide [13] which is a marine-derived macrolide isolated from deep-water sponges collected off Jamaica’s north coast of the family Neopeltidae (Wright et al., 2007a).

Neopeltolide is a highly cytotoxic and potent inhibitor of tumor cell proliferation *in vitro* at nanomolar concentrations. However, it may act as a cytostatic agent depending on the dose, and also displays powerful antifungal action (Wright et al., 2007a). The mechanism of action is still under research, but it has been suggested that neopeltolide does not act via interaction with tubulin or actin (Wright et al., 2007a). In addition, it inhibits mitochondrial adenosine triphosphate (ATP) synthesis by targeting the cytochrome bc_1_ complex (Ulanovskaya et al., 2008).

Given the potent antiproliferative activity of neopeltolide in cancer cells, efforts to develop drugs based on this lead were evident in the last two decades (Bai and Dai, 2015). One barrier to this ambition was however the scarcity of natural sources which initiated another line of research on the synthesis. This review aims to summarize recent advances in neopeltolide and its analogues as anticancer agents with emphasis on their existing medical applications, the knowledge gaps and opportunities and future directions.

## 2 Review methodology

A review was conducted on the web (Scopus, Google Scholar, and PubMed) to cover all relevant publications until March 2023. Generally, the following words and Boolean operators were used, “neopeltolide,” “potential anticancer studies AND *in vitro* OR *in vivo*,” “medical applications,” “mechanism of antitumor action,” and “marine-derived macrolide.” All the articles that were included were in English. The main findings on the biological activity of neopeltolide were recorded by recording cell lines used, IC_50_ or LD_50_, and the main outcomes. Other data on the biological activity of neopeltolide *in vitro* or those obtained through molecular docking were also included.

## 3 Structural characterization of neopeltolide

Neopeltolide is a natural marine product isolated and purified from a sponge of the *Lithistida* group, corresponding to the Neopeltidae family. It was reported for the first time by Wright et al., in 2007 from two specimens obtained from a rocky outcrop at a depth of 442 m on the northwest coast of Jamaica. The specimens were closely related to the genus *Daedalopelta Sollas* (Wright et al., 2007a).

**FIGURE 1:**
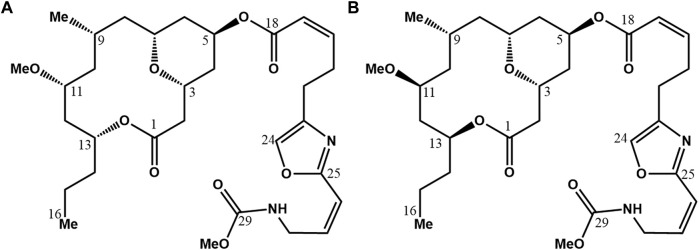
Neopeltolide structure proposed by Wright et al. (2007a). **(A)** And the revised structure by Youngsaye et al. (2007) and Custar et al. (2009) **(B)**.

Structurally, neopeltolide (Figure 1) is a macrolide composed of a 14-membered macrocyclic lactone ring with an ether bridge between C3 and C7, and a 2, 4, 6-trisubstituted tetrahydropyran moiety. Tetrahydropyrans are biologically relevant structural motifs abundantly present in marine natural products. At C5, the hydroxyl group is acylated in the axial position on the pyran ring, with a side chain containing oxazole and carbamate groups; this side chain is identical to that of (+)-leucascandrolide A (D'Ambrosio et al., 1996). Neopeltolide’s structure features six stereogenic centers. Initially, Wright (Wright et al., 2007a) put forth the relative configuration of neopeltolide upon isolating it (Figure 1A). Subsequently, Panek (Youngsaye et al., 2007) and Scheidt (Custar et al., 2009) independently synthesized the proposed and accurate structures, determining the absolute molecular configuration and designating the S configuration for stereogenic centers C11 and C13 in (+)-Neopeltolide (Figure 1B).

Nowadays, neopeltolide can be obtained through total synthesis via several methodological approaches; all require an efficient and stereoselective construction of the tetrahydropyran rings. More than twenty studies have described various synthetic strategies, including Prins reaction and its variants, the hetero-Diels-Alder cycloaddition of Danishefsky’s, ring-closing metathesis, intramolecular radical cyclizations, intramolecular oxa-Michael reaction, Palladium-catalyzed intramolecular alkoxycarbonylation, transannular oxymercuration, and desymmetrizing ring-opening/cross-metathesis cascade (Fuwa, 2016).

There is a lack of information about neopeltolide’s physical and physicochemical characteristics: It has a molecular weight of 590.7 g/mol and, after purification, is obtained as a colorless oil with ${\left[\alpha \right]}_{D}^{24}$ = +24(c 0.24, MeOH). Also, analogs are obtained as colorless to yellow oils (Cui et al., 2010). The liquid state can make it challenging to formulate neopeltolide into dosage forms. In contrast, parenteral administration, such as intravenous or intramuscular route, is preferred for the clinical application of neopeltolide after dilution into the appropriate vehicle. Until now, no studies have been directed to modify the physical state of neopeltolide. Relevant properties of neopeltolide, such as Log P (4.7) and Hydrogen Bond Acceptor Count, have been computed; however, the water solubility of neopeltolide, melting point, and chemical stability have not been reported.

Neopeltolide is a potent inhibitor of tumor cell proliferation *in vitro*. After a careful study, it was possible to identify the pharmacophoric groups and their configurations. According to (H. Fuwa et al., 2013), the high antiproliferative activity is related to oxazole and methyl carbamate moieties in the side chain. In addition, the C5 oxazole must be arranged axially on the tetrahydropyran ring, and these two elements constitute the minimum structural requirements for the activity. Other factors that contribute to potentiating the antiproliferative activity are configuring the C11 and C13 stereogenic centers. Their configuration is essential to define the general shape of the macrocyclic skeleton. In further studies by the Sasaki group, the roles of double bonds within the oxazole-containing side chain and the terminal methyl carbamate group have been established. The authors also reported that the C19–C20 and C26– C27 double bonds and the terminal methyl carbamate group are indispensable structural elements for low nanomolar antiproliferative activity (Fuwa et al., 2014a).

## 4 Description of neopeltolide in official sources

**FIGURE 2:**
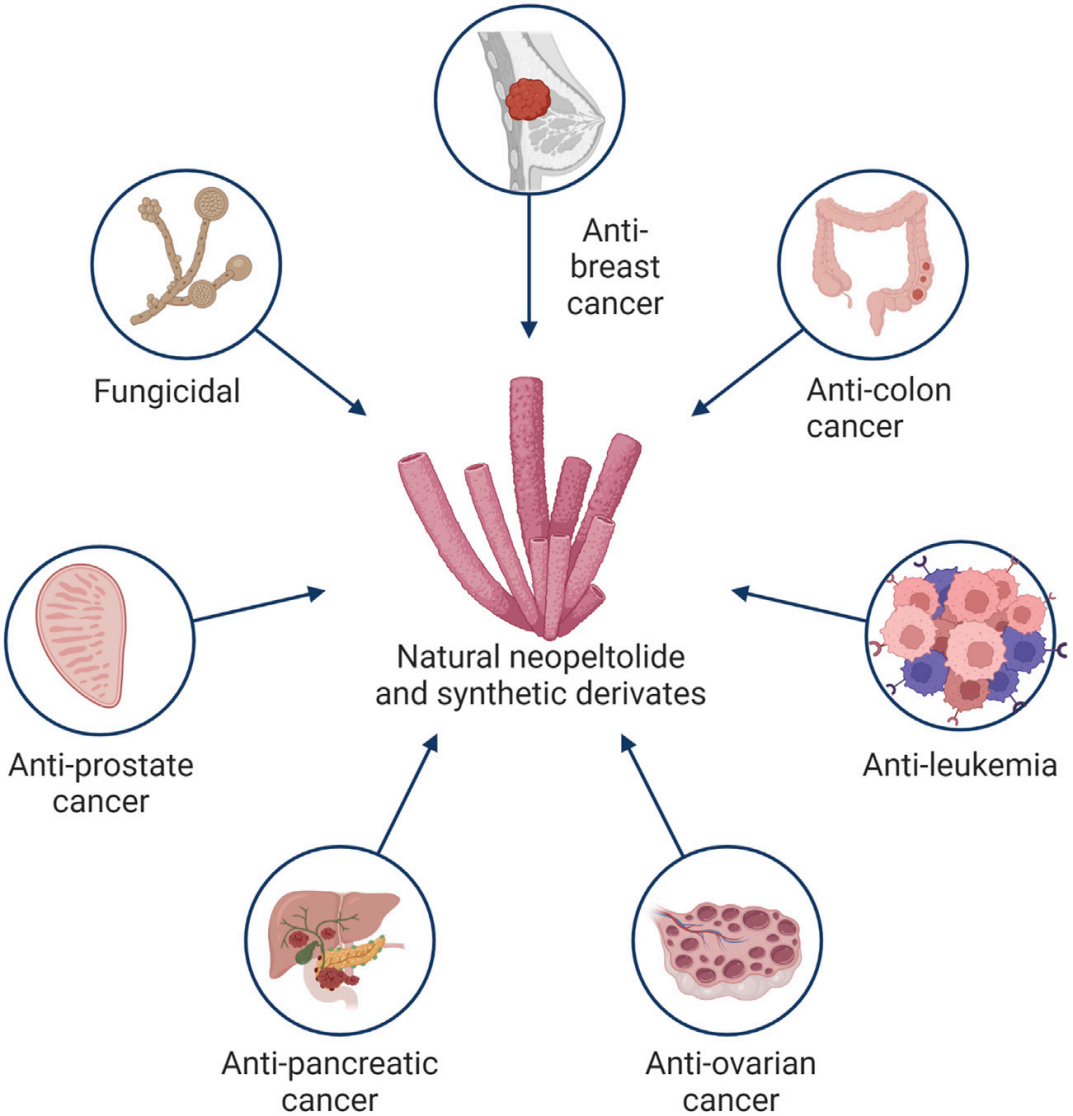
Potential applications of the natural extract neopeltolide and its synthetic derivates.

To the best of our knowledge, there is no record of neopeltolide in pharmacopeias, much less any classification by international organizations such as WHO. Neopeltolide is a natural product from which other synthetic products are derived; however, it is not an official drug, so it is not currently included in the pharmacopoeia, national formulary, or pharmaceutical code. As a non-official drug, neopeltolide is published in journals where the biological value is demonstrated through preclinical tests. Still, more knowledge of various molecular and biopharmaceutical properties must be gained. Information from official sources comes from the U.S. Patent and Trademark Office, with the patent registration US20040266847A1 assigned to Harbor Branch Oceanographic Institution Inc. on 18 August 2004, and later 14 August 2009, as US7179828B2 assigned to the National Institutes of Health (NIH), U.S. Department of Health and Human Services (DHHS), U.S. Government. The patent has an adjusted expiration date of 7 July 2025 (Wright et al., 2007b). The primary purpose of invention established by the inventors Amy E. Wright, Shirley A. Pomponi, and Peter J. McCarthy includes the extraction compounds, analogs, and their pharmaceutical formulations for anticancer use in cells of the breast, colon, central nervous system, ovarian, renal, prostate, liver, pancreatic, uterine, lung tumor, leukemia, and melanoma. Also, the invention is assigned for use in controlling fungal growth as fungicidal, fungistatic, and inhibition of fungal germination applied in plant and animal fungal infections and possible preservatives in food and cosmetics (Wright et al., 2007b). Some of the potential applications of neopeltolide and its derivates are shown in Figure 2. The invention describes the collection of a sponge at latitude 18 28.638′N, longitude 78 10.996′W at a depth of 433 m. The inventors have a reference sample preserved in ethanol in the Harbor Branch Oceanographic Museum (catalog number 003:01004, DBMR number 23-VIII-93-5–010). After extensive traditional extraction procedures, the inventors do not mention the production yield. The first reports of cytotoxicity were established at IC_50_ for A549, NCIADR-RES, and P388 at 1.17, 5.1, and 0.56 nM, respectively. With a minimum inhibitory concentration of neopeltolide as a fungal growth inhibitory against *C. albicans* of 0.625 μg/mL (Wright et al., 2007b). Interestingly, the disclosure of the invention, as a common strategy in the extension of information protection, includes a “Formulation and administration” section where there is no convenient formulation for new compounds and only the suggestion of possible conventional pharmaceutical dosage forms. Currently, pharmaceutical systems are preferred and sought for anticancer molecules that allow a certain degree of vectorization to minimize adverse effects and resistance phenomena and therefore increase the efficiency of drug treatment. In the invention, the dimethyl sulfoxide diluent suggested for neopeltolide is currently prohibited, even in preclinical trials in animal models. Finally, the invention suggests, in an “illustrative” manner, some dosage ranges for different routes of administration. Unfortunately, as sporadically mentioned in the same invention, the dosage will depend on the type of living organism, pathology, route of administration, and pharmaceutical form. In the first instance, the racemic purity must be carefully monitored; in the second instance, the cytotoxicity assays must ensure the safety of living organisms, then further exploration of the pharmacology of neopeltolide from pharmacodynamics to pharmacokinetic assays to then establish the appropriate pharmaceutical forms and convenient route of administration for each type of cancer. Then, biopharmaceutical studies would determine the suitability of administration. Finally, the National Library of Medicine, a National Center for Biotechnology Information from the National Institutes of Health, U.S. Department of Health and Human Services, U.S. Government, includes a brief description of the invention “biologically active neopeltolide compounds” on its website (National Center for Biotechnology Information, 2023). Likewise, the Food and Agriculture Organization of the United Nations portal includes a brief description of neopeltolide from the National Agricultural Library of the United States (Athe et al., 2012).

## 5 Synthetic derivatives

**FIGURE 3:**
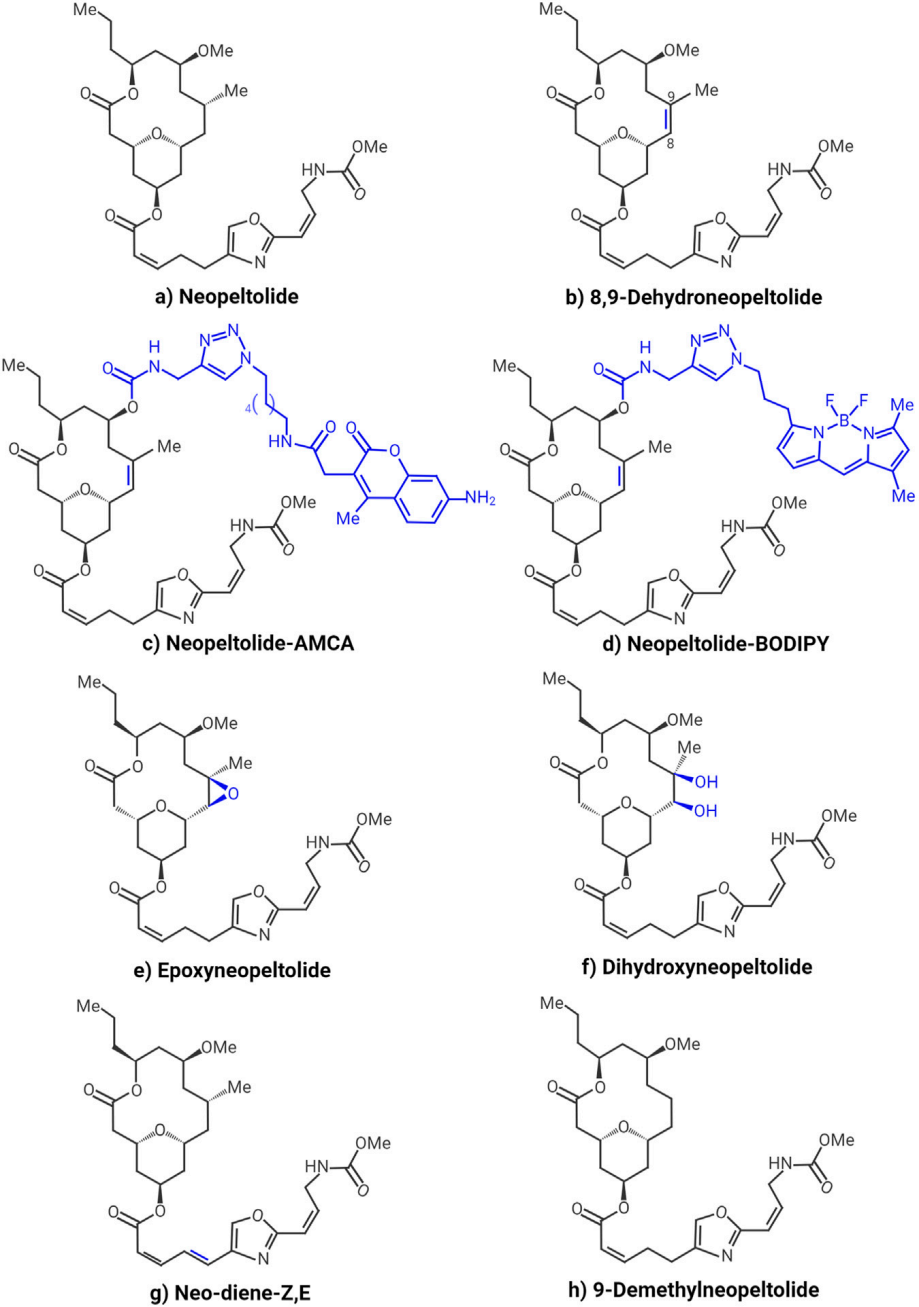
Mainly synthetic derivatives of neopeltolide with anticancer potential: Neopeltolide **(A)**, 8,9-Dehydroneopeltolide **(B)**, Neopeltolide-AMCA **(C)**, Neopeltolide-BODIPY **(D)**, Epoxyneopeltolide **(E)**, Dihydroxyneopeltolide **(F)**, Neo-diene-Z,E **(G)** and 9-Demethylneopeltolide **(H)**.

Neopeltolide is a structurally and biologically interesting molecule as a lead for synthesizing anticancer drugs. It induces potent antiproliferative activities *in vitro* in several cancer cell lines, including the A549 human lung adenocarcinoma cell line, the P388 murine leukemia cell line, the MCF-7 human breast cancer cell, the HCT-116 human colorectal carcinoma cell line, and the PANC-1 human pancreatic carcinoma cells (Cui et al., 2012; Fuwa et al., 2014a). These effects have been found at nanomolar concentrations, underpinning their potencies. Various strategies have been developed for synthesizing neopeltolide and its derivatives to explore the structure-activity relationship and identify more potent compounds with better biopharmaceutical properties. Fuwa et al. demonstrated that the tetrahydropyran ring attached to the oxazole-containing side chain is the essential for the observed biological activity (Fuwa et al., 2013). This same group performed the synthesis and biological evaluation of neopeltolide analogs with structural modifications in the oxazole-containing side chain. The authors evaluated the antiproliferative activity against A549 and PANC-1 cells. They found that the C19-C20 and C26-C27 double bonds within the oxazole-containing side chain and the terminal methyl carbamate group are indispensable structural moieties for the antiproliferative activity of neopeltolide (Fuwa et al., 2014a). Therefore, at least the tetrahydropyran ring, C19-C20 and C26-C27 double bonds, and methyl carbamate should remain unchanged in synthetic neopeltolide derivatives as potential anticancer agents. Also, Cui et al. synthesized neopeltolide (Figure 3A) and various analogs through C8-C9 alkene functionalization and side chain variation. The authors evaluated growth inhibition against MCF-7 and HCT-116 and found that structural alteration of the C8-C9 domain is possible without a significant loss in anticancer potency (Cui et al., 2012). Although neither neopeltolide derivative resulted in increased potency, it was established that retention of the alkene group required for oxidative cyclization leads to a potent analog (8,9-dehydroneopeltolide, Figure 3B) that can be prepared in fewer steps than the natural product (Cui et al., 2012). On the other hand, it has been shown that this analog is up to 3 times more active against the A549 cell line (Fuwa et al., 2013). The 8,9-dehydroneopeltolide analog appears to be highly promising, as one study revealed that it induces apoptotic cell death in HL-60 human promyelocytic leukemia cells under energy stress conditions and can induce non-apoptotic cell death in the presence of zVAD (pan-caspase inhibitor) (Fuwa et al., 2014b). Yanagi et al. (Yanagi et al., 2019) synthesized and analyzed fluorescent derivatives of neopeltolide. Since the proper choice of fluorophore is critical to successful live cell imaging using fluorescent derivatives of natural products, the authors tested two fluorophores of different volumes and lipophilicity: 7-amino-4-methyl-coumarin (AMCA) and boro-dipyrromethene (BODIPY). Moreover, the researchers evaluated the biological activity of the resulting derivatives. The antiproliferative activity was examined *in vitro* in A549 cells; the IC_50_ for neopeltolide was 0.68 nM, for neopeltolide-AMCA (Figure 3C) 1.5 μM, and for neopeltolide-BODIPY (Figure 3D) 63 nM. Although the derivatives were slightly less active than neopeltolide, these data indicated that they still had sufficient biological activity and would be useful for live cell imaging (Yanagi et al., 2019). On the other hand, neopeltolide has been synthesized through a brief sequence that highlights using ethers as oxocarbenium ion precursors. Some key steps include an acid-mediated etherification and a sequence featuring a Sonogashira reaction, an intramolecular alkyne hydrosilylation reaction, and a Tamao oxidation. The alkene that is required for oxidative cyclization can be hydrogenated for the synthesis of the natural product (neopeltolide), or it can be epoxidized (epoxyneopeltolide, Figure 3E) or dihydroxylated (dihydroxyneopeltolide, Figure 3F) to obtain more polar derivatives (Cui et al., 2010). Vintonyak performed the synthesis of neopeltolide derivatives by coupling oxazole-containing acids with the neopeltolide core (neo-macrolactone) and obtained a compound (neo-diene-ZE, Figure 3G) that, according to cell assays, it is almost twice as active as neopeltolide itself (IC_50_ 0.16 nM vs. 0.25 nM). This potentiation, in effect, can be explained by restricting an original flexible bond between the oxazole ring and the Z-enoate moiety by introducing an additional double bond, which leads to the fixation of a more active conformation (Vintonyak et al., 2008a). Another interesting neopeltolide derivative is 9-demethylneopeltolide (Figure 3H), first synthesized by Fuwa et al. by exploiting a Suzuki-Miyaura coupling/ring-closing metathesis strategy (Fuwa et al., 2009) and later obtained by other techniques such as palladium-catalyzed intramolecular alkoxycarbonylation for the simultaneous construction of the tetrahydropyran ring and the macrocyclic skeleton (Bai et al., 2014). The evaluation of the antiproliferative activity against P388 revealed that this compound is equipotent or slightly more active than neopeltolide (IC_50_ 0.899 nM), exhibiting cytotoxicity with IC_50_ of 0.813 nM. Although the potency was not significantly increased due to its simplified structure and synthetic accessibility, it is an attractive starting point for detailed investigations into structure-activity relationships and biological activities of neopeltolide (Fuwa et al., 2009). The chemistry developed in these neopeltolide derivatives exemplifies the structure’s ability to be modified, aiming to retain or increase the biological potency and/or improve the accessibility to these compounds.

## 6 Current medical applications—official treatment or traditional medicine

In recent years, the search for secondary metabolites in marine organisms revealed a range of novel natural products with interesting structures and biological activities (Vintonyak et al., 2008b) that are potentially useful for dissecting complex cellular biological events at the molecular level. In this sense, several marine natural products have recently been developed into clinically approved drugs, including trabectedin and eribulin mesylate, and more are currently under clinical trials (Fuwa, 2016). Although thousands of chemical compounds from sponges have been reported in the literature, only a few are clinically described. Many studies revealed that sponge-derived metabolites are used directly in therapy or as a prototype of bioactive leads to develop more active and less toxic analogs (Musarat and Nawal, 2018).

As described above, neopeltolide exhibits a potent cytotoxic effect against different cancer cell lines and fungal strains (Wright et al., 2007a; Cui et al., 2010). In addition, Marine sponges of the polyphyletic order “*Lithistida*” have been the source of a wealth of natural products with various biological activities (Yang et al., 2011).

Concerning its antifungal properties, neopeltolide is a potent inhibitor of the growth of pathogenic fungi, *Candida albicans*, which severely and adversely affects the health of AIDS patients, potentially resulting in death. Based on the study conducted by Amy E. Wright et al., neopeltolide presented a growth inhibitory zone of 17 mm when tested at a concentration of 25 μg/disk in the *C. albicans* disk diffusion assay and a minimum inhibitory concentration (MIC) in liquid culture of 0.625 μg/mL (Wright et al., 2007a). Since the first publication by Wright et al. about the discovery of neopeltolide, there has been no evidence of further exploration of the compound in traditional medicine. There are no registered clinical trials or official treatments. Part of this timeline is associated with the lack of knowledge of the compound’s major physical and chemical characteristics, the extremely restricted source of obtaining it, and the lack of economically accessible analogs. However, preliminary trials offer a promising outlook in the search for new alternatives in treating different types of cancer.

## 7 Potential anticancer studies

Although at least half of the current medical prescriptions are based on drugs derived from natural sources (Newman and Cragg, 2020), the relevant biological activities of natural products are still challenging to analyze and locate molecular targets. This translates into a challenge in developing and applying pharmaceutical formulations that can be applied in clinical practice (Huang et al., 2021). Initially, studies focus on molecular characterization and *in vitro* evaluations, followed by *in vivo* and clinical phase trials to ensure that new drugs are effective and safe for widespread use (Atanasov et al., 2021). This section discusses the most relevant studies of neopeltolide to date and their status (Table 1).

**TABLE 1:** Main findings on the biological activity of neopeltolide.

Cellular line	IC_50_	Main results	References
A549 human lung adenocarcinoma	1.2 nM	Block of the cell cycle Not act via tubulin or actin interactions	Wright et al. (2007a)
NCI/ADR-RES ovarian carcinoma	5.1 nM		
P388 murine leukemia	0.56 nM		
A549	0.3–0.5 nM	Inhibition of cytochrome *bc*_*1*_	Ulanovskaya et al. (2008)
PC3			
HCT116			
Cervical cancer tissue		Inhibition of SLC30A10 gene	Zhang et al. (2022)
L929	0.25 nM	E-isomers of neopeltolide are less active	Vintonyak et al. (2008b)
HCT116-p53KO	9.9	Neopeltolide and its analogs have a selective cytotoxic effect	Cui et al. (2012)
P388 murine leukemia	10.1	Structural effects over cytotoxic activity	Fuwa et al. (2009)
A549	0.5	Data for the analog (−)-8,9-dehydroneopeltolide	Fuwa et al. (2014a)
PANC-1	2.4		
**Complex bc1**			
Porcine SCR (mixture of bc_1_ complex with complex II)	0.74–1.75 *μ*M	Structures designed by molecular docking	Xiong et al. (2020)

### 7.1 *In vitro* studies

The first studies of cancer cell antiproliferative activity of neopeltolide were generated after observing the ability to inhibit the growth of *C. albicans,* and the first mechanism of action was stated by Ulanovskaya et al. (Ulanovskaya et al., 2008). Wright et al. further found that neopeltolide could effectively inhibit A549 human lung adenocarcinoma, NCI/ADR-RES ovarian sarcoma, and P388 murine leukemia. However, when neopeltolide interacts with p53 mutated lines such as PANC-1 pancreatic cancer and DLD-1 colorectal adenocarcinoma, it exhibits a cytostatic rather than cytotoxic effect. Therefore, these experiments concluded that the antitumor action did not come from the interaction with tubulin or actin (Wright et al., 2007a).

Wright et al., found that structurally neopeltolide is a simplified version of leucascandrolide A (Wright et al., 2007a), and Ulanovskaya and coworkers began a series of studies to compare the mechanism of action and efficacy of each in inhibiting cell proliferation (Ulanovskaya et al., 2008). Then, in 2008 (Vintonyak et al., 2008b), reported the inhibition of breast cancer cell lines (L929) and validated the previous findings for the A549 cell line. In this study, the authors found from a series of structural modifications that the E-isomers of neopeltolide are less active (Fuwa et al., 2009). demonstrated the possibility of simplifying the original structure of neopeltolide without affecting its biological activity, which was confirmed in P388 murine leukemia cells. The authors found that it is possible to reduce the necessary dose with structural modifications in the C11 methoxy and C9 methyl groups. The researchers also observed that the macrocycle or the oxazole subunit presented biological activity, and they are indispensable components to producing a cytotoxic effect.

Also (Custar et al., 2009), identified that the antitumor action of neopeltolide is selective because, although the authors registered an inhibitory effect on MCF-7 and P388 lines, they did not observe any cytotoxic effect on human cervical carcinoma lines HeLa, rat adrenal tumor PC12, human epidermal carcinoma KB and human lung carcinoma A549. In 2012, Cui et al. (2012) synthesized several analogs and observed the growth inhibition in different cell lines, such as MCF-7, HCT-116, and HCT-116 cell lines without p53, concluding that neopeltolide and its analogs have a selective cytotoxic effect.

Cui et al. (2012) also found that modifications at the C8- and C9- positions do not affect the biological activity of neopeltolide, whereas retaining the alkene group necessary for oxidative cyclization results in a potent analog, 8,9-dehydroneopeltolide that can be prepared in one step less than the natural product, while hydroboration of the alkene produces an active analog with higher polarity. In addition, the authors identified that side chain modifications significantly diminish the cytotoxic effect, an exception being when the furan group replaces the oxazole group. The biological activity results indicated that the presence of p53 in the cells is indispensable to achieving the desired effect.

In another research from Fuwa’s laboratory studying the relationship of chemical structure to biological activity, the authors tested the antiproliferative activity of a series of analogs on A549 and PANC-1 cell lines. They found that the C19-C20 and C26-C27 double bonds and the methyl carbamate end group were essential to keep the lethal dose below nanomolar concentration (Fuwa et al., 2014a).

In 2014, Fuwa et al. (2014b), taking up their previous synthetic work and that of other researchers, decided to test the biological effect of the primary neopeltolide derivative, (−)-8,9-dehydroneopeltolide (8,9-DNP), a synthetically more accessible analog. The cytotoxic effect of this compound was tested under normal and stress conditions. These experiments concluded that the analog induced apoptotic death of HL-60 human promyelocytic leukemia cells and enhanced this effect under stress conditions generated by nutrient deprivation, such as glucose.

Due to these findings, Fuwa and Sato (2017) studied this phenomenon in depth, as cells within a tumor microenvironment are often under hypoxia and nutrient deficiency conditions due to the high energy demand required for excessive cell proliferation and vascularization. In that context, these cells even incur amino acid recycling and autophagy of cytoplasmic components to generate energy. Then, the cytotoxicity of this analog was tested in starved pancreatic adenocarcinoma PANC-1 cells and non-small cell lung adenocarcinoma A549 cells. Due to contact with 8,9-DNP, these cells were deprived of energy sources and underwent necrotic death. Thus, 8,9-DNP is a potent anti-austerity agent that impairs mitochondrial ATP synthesis and cytoprotective autophagy in starved tumor cells, a behavior relevant to cancer treatment.

In 2019, Zhu et al. (2019) identified neopeltolide as a Q_o_ site inhibitor through extensive molecular docking, molecular dynamics simulations, and Poisson-Boltzmann surface molecular mechanics calculations. Using these tools, the authors concluded that the site inhibition was due to the formation of hydrogen bonds between several critical points of the bc_1_ complex. Considering the information obtained, the authors synthesized a series of neopeltolide analogs, finding one of them ((*Z*)-4-((7-bromonaphthalen-2-yl)-oxy)benzyl-2-(3- ((methoxycarbonyl)amino)prop-1-en-1-yl)oxazole-4-carboxylate) with high potency to inhibit porcine SCR with an IC_50_ of 12 nM.

In 2020, Xiong et al. (2020) synthesized a series of neopeltolide derivatives by replacing the 14-membered macrolactone with an indole ring. Based on the molecular docking and binding free interactions energy calculations with the bc_1_ complex, the IC_50_ values of several derivatives ranging from 0.70 to 1.46 µM were significantly improved by replacing the ester with an amide linker. Subsequently, with computational molecular docking studies, the researchers found that the potent binding affinity and inactivation activity of the bc_1_ complex was related to the formation of hydrogen bonds and *π*-π interactions with the synthesized derivatives. From these elucidations, computationally designed derivatives could be obtained by optimizing the biological activity. In 2022, Zhang et al. (2022) identified that neopeltolide could inhibit the high expression of the SLC30A10 gene, which belongs to the ZnT gene family that is strongly related to tumor development and metastasis.

Despite all the *in vitro* studies that have demonstrated the high cytotoxic effects of neopeltolide and its derivatives against various cancer cell lines, there is still no *in vivo* evidence that allows taking the next step towards a clinical evaluation. This may be due to the difficulty of obtaining this natural product synthetically. So far, most reports are based on the comparative study of the different structural modifications and their implication in altering biological activity. However, more research is needed to understand these compounds’ effects and possible applications in animal models.

## 8 Mechanism of antitumor action of neopeltolide

Antitumor agents prevent or inhibit the formation or growth of tumors and are known as antitumor, anticancer, chemotherapeutic, or antimetastatic agents. Antitumor agents kill those cells that divide rapidly, which is one of the main properties of most cancer cells. Besides this characteristic, these cells more readily use glycolysis, an inefficient metabolic pathway for energy metabolism, even when sufficient oxygen is available (Penta, 2016). This dependence on aerobic glycolysis stimulates tumorigenesis and malignancy progression.

In normal cells, the mitochondrial electron transport chain (mETC) is responsible for 90% of the ATP synthesis while in tumoral cells, it contributes to about 50% of the total ATP level (Penta, 2016; Raimondi et al., 2020). Despite this, inhibition of mETC is an important cancer therapeutic target. The mETC comprises four multi-protein complexes (I-IV) inserted in the inner membrane. Complex I and II oxidize NADH and FADH_2_, transferring the resulting electrons to ubiquinol, which carries electrons to Complex III. Complex III pushes the electrons across the intermembrane space to cytochrome c (Cyt c), which brings electrons to complex IV. Complex IV employs these electrons to reduce oxygen to water (Guo et al., 2018; Nolfi-Donegan et al., 2020).

Complexes I, II, and III generate superoxide: Complex I and II generate ROS inside the mitochondrial matrix, and Complex III generates ROS in both the matrix and the intermembrane space. Superoxide generated in the intermembrane space can escape into the cytoplasm through voltage-dependent anion channels.

**FIGURE 4:**
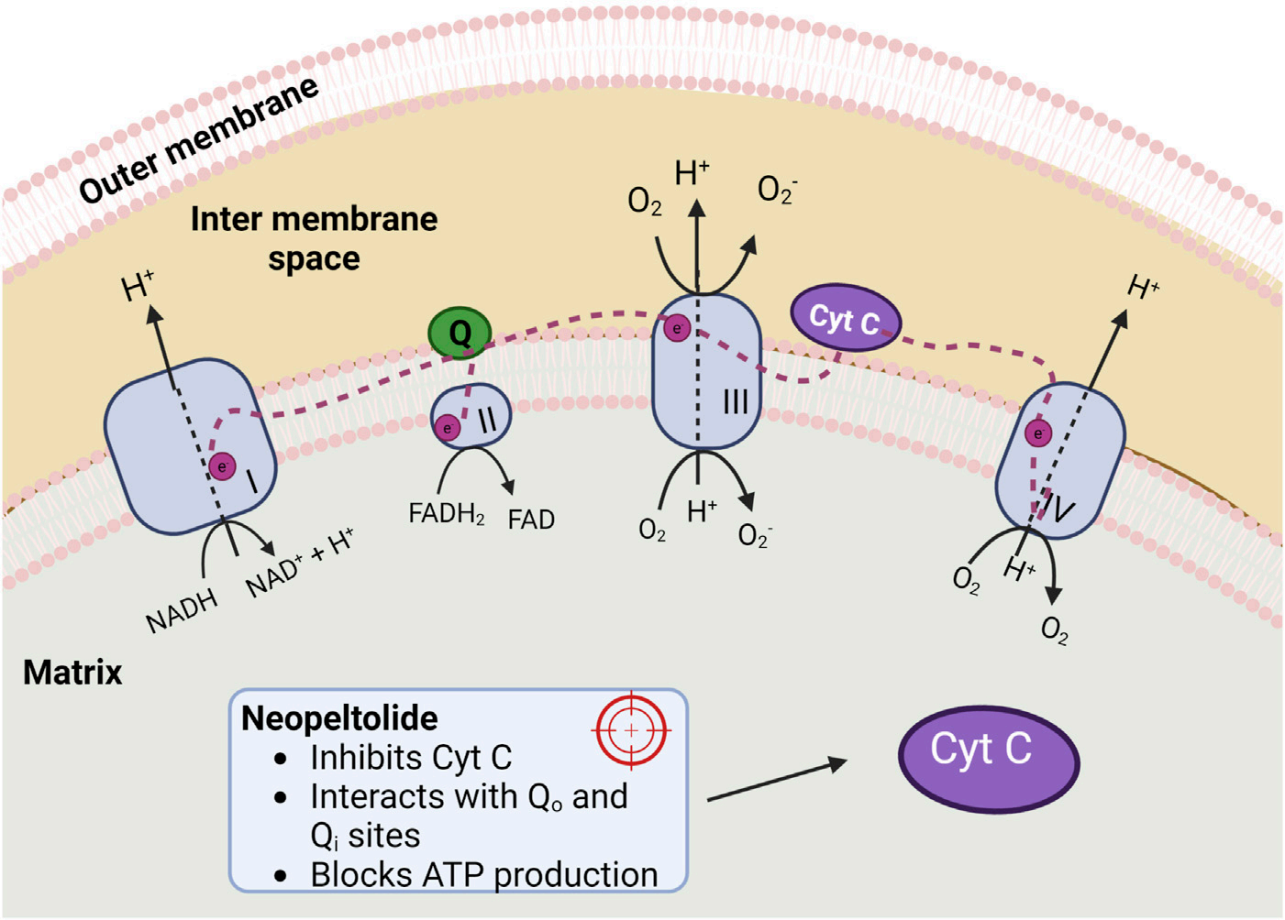
Four complexes of the mitochondrial electron transport chain. Complex I (NADH-coenzyme Q reductase), Complex II (succinate dehydrogenase), Complex III (coenzyme Q-cytochrome c reductase), and Complex IV (cytochrome c oxidase). Neopeltolide inhibits cytochrome c (Cyt C), decreasing ATP production.

Neopeltolide is reported to inhibit Complex III (cytochrome bc1 complex) of the mETC (Ulanovskaya et al., 2008; Yanagi et al., 2019; Xiong et al., 2020) (Figure 4) suppressing ATP synthesis. The bc_1_ complex is a multi-subunit enzyme in the mid-segment of the mitochondria’s cellular respiratory chain. In general, cytochromes bc are present in almost every living cell, representing one of the broadest groups of energy-transducing enzymes (Xia et al., 2013). They are critical components of both respiratory and photosynthetic electron transport chains, and their function is essential for the function of these chains.

The bc_1_ complex catalyzes the reaction of transferring electrons from the low-potential substrate ubiquinol (hydroxyquinones, QH_2_) to high-potential Cyt c (Sarewicz et al., 2021). Additionally, bc_1_ translocates protons across the membrane, contributing to the proton-motive force essential for various cellular activities such as ATP synthesis. The core of the bc1 complex contains two binding sites according to the Q-cycle reaction mechanism: the quinone reduction site (Q_i_) and the quinol oxidation site (Q_o_), which are distant from each other by over 30 Å (Cooley et al., 2009; Sarewicz et al., 2021). The first one is close to the negative side of the membrane, while the Q_o_ site is close to the positive side. If the electron transport process of the bc_1_ complex is disturbed in both sites, the cell will die due to the blocking of ATP production.

Recent reports analyzed by docking how neopeltolide interacts with Q_o_ and Q_i_ sites, showing that the neopeltolide-Q_o_ complex is more favorable than neopeltolide-Q_i_ due to the first exhibiting a better binding energy (−9.44 kcal/mol) than the second one (−8.90 kcal/mol). These results were confirmed by MD simulations indicating that neopeltolide should bind to the Q_o_ site of the bc1 complex (Zhu et al., 2019; Xiong et al., 2020).

Due to the biomedical importance of this molecule, further studies should be done to have a deeper understanding of its mechanisms.

## 9 Conclusion

Although cancer mortality has declined over the last three decades, it is still the most significant disease that causes death in most countries. Hence, there is a continued interest in developing effective natural medicines that decrease the side effects, drug resistance, and organ toxicity produced by synthetic drugs used for chemotherapy in cancer treatment. Sponges are marine animals rich in bioactive natural products. The isolation neopeltolide from a marine sponge and its anticancer properties, such as cytotoxicity and the inhibition of tumor cell proliferation, were first reported in 2007. Thus, neopeltolide represents a potentially promising novel drug for cancer therapy.

Based on this background, the present review summarized the principal factors related to the structural attributes of neopeltolide and scrutiny of the available literature on its antitumor effects. We identified three central challenges about this topic: 1), the effective dose of neopeltolide as an anticancer compound; 2), limited availability of the compound to do *in vivo* studies; and 3), the necessity to establish the absolute stereochemistry of neopeltolide to better predict its molecular mechanism of action. Significant advances have been made in neopeltolide synthesis and in testing the effects of neopeltolide in cells. However, efforts are still required to advance clinical applications and their use as chemotherapeutics.

## Abbreviations

AIDS, Acquired immunodeficiency syndrome; AMCA, 7-amino-4-methylcoumarinbc1 complex: Cytochrome bc1 complex; BODIPY, Boro-dipyrromethene; Cyt c, Cytochrome c; 8,9-DNP, (-)-8,9-dehydroneopeltolide; IC_50_, Inhibitory concentration; mTEC, Mitochondrial electron transport chain; WHO, World Health Organization.
